# Being kind to ourselves: group compassion-focused therapy (CFT) versus treatment as usual (TAU) to improve depression and anxiety in dementia – a protocol for a mixed-methods feasibility randomised controlled trial within the NHS

**DOI:** 10.1136/bmjopen-2024-093249

**Published:** 2024-12-03

**Authors:** Aimee Spector, Melissa Melville, Catriona Craig, Catherine Henderson, Syd Hiskey, Martin Knapp, Yvette Kusel, Keith Oliver, Louise Robinson, Lindsay Royan, Joshua Stott, Lisa Williams, Rachel Evans

**Affiliations:** 1 Department of Clinical Health Psychology, University College London, London, UK; 2 Research and Development Department, North East London NHS Foundation Trust Goodmayes Hospital, Ilford, UK; 3 Buckinghamshire's Older People's Psychological Services, Saffron House, Easton Street, High Wycombe, Oxford Health NHS Foundation Trust, Buckinghamshire, UK; 4 Care Policy and Evaluation Centre, The London School of Economics and Political Science, London, UK; 5 Private Practice, The Oaks Hospital, Colchester, UK; 6 Kent and Medway NHS and Social Care Partnership Trust, Thanet Older Adult Psychology, The Beacon, Manston Road, Ramsgate, UK; 7 Alzheimer's Society Ambassador, London, UK; 8 Newcastle University Population Health Sciences Institute, Newcastle upon Tyne, UK; 9 Clinical Health Psychology Services, North East London NHS Foundation Trust, London, UK; 10 Adapt Lab, Research Department of Clinical, Educational and Health Psychology, University College London, London, UK; 11 Mersey Care NHS Foundation Trust, Liverpool, UK; 12 School of Health Sciences, Bangor University, Bangor, UK

**Keywords:** Dementia, Anxiety disorders, Depression & mood disorders, Feasibility Studies, Psychosocial Intervention

## Abstract

**Introduction:**

Depression and anxiety are common in dementia, with a devastating impact. However, there remains a lack of evidence-based psychological therapies for this clinical group. Compassion-focused therapy (CFT), a talking therapy which addresses feelings of shame and stigma, has shown benefits in other clinical populations. This study is a mixed-methods feasibility randomised controlled trial (RCT) of group CFT for people with dementia and symptoms of anxiety and/or depression, to determine if a future definitive RCT is feasible.

**Methods and analysis:**

50 people with mild-to-moderate dementia and symptoms of anxiety and/or depression will be randomised to either the intervention arm (12 sessions of group CFT plus treatment as usual (TAU)) or the control arm (TAU). Primary outcome measures include the feasibility of conducting an RCT in terms of recruitment, acceptability, suitability of secondary outcome measures and fidelity. Blind assessments will be conducted at baseline, approximately 16 weeks and 6 months follow-up, to collect data on depression, anxiety, quality of life, quality of the carer–patient relationship, cognition, self-compassion and carer burden. Qualitative interviews will be used to gather participant, carer/supporter and clinician perspectives on the value, acceptability and feasibility of the intervention.

**Ethics and dissemination:**

This study has been approved by the London Riverside REC (Ref: 23/LO/0535) and the Health Research Authority (HRA) ethical approval process through the Integrated Research Application System (IRAS ID: 327086). We plan to publish the results in open-access peer-reviewed journals, present data at conferences and provide feedback to the study participants, sponsors and funders.

**Trial registration number:**

ISRCTN20868432.

**STRENGTHS AND LIMITATIONS OF THIS STUDY**

- A strength is the mixed-methods approach, enabling triangulation of information to enhance the reliability and validity of our findings.
- Given that people with dementia might lack technology, Wi-Fi, transport provision or have comorbid health or mobility problems, a strength of this study is offering the choice of virtual or face-to-face attendance.
- A strength is our inclusive approach, recruiting from an underserved research area, Barking and Dagenham, which has the highest ‘Index of Multiple Deprivation’ score in London and 21/317 in England.
- A potential limitation is the availability of psychologists to deliver the intervention, preventing some Trusts from being able to take part as research sites.

## Introduction

 Depression and anxiety are common in dementia and can have a devastating impact including accelerated cognitive decline, increased need for institutional care and premature death. There are currently no pharmacological or psychological therapies with established efficacy for these individuals, presenting a critical gap in both treatment and care. A meta-analysis^1^ suggested that psychosocial treatments for both depression and anxiety in people with dementia are limited, but they provide an important avenue of likely benefit.

Compassion-focused therapy (CFT)^2^ is a talking therapy that integrates techniques from evolutionary, social and developmental psychology. It focuses on reducing self-criticism and shame, acknowledging the emotional impact of difficult experiences, building compassion for the self and others and facilitating adjustment and acceptance. The theoretical stance lends itself well to those with mild-to-moderate dementia, for whom stigma can result in shame, embarrassment and self-criticism.^3^ CFT specifically addresses how people with dementia respond to their cognitive deterioration. Developing acceptance is likely to facilitate adjustment and be emotionally protective, ultimately reducing clinical depression and anxiety and improving well-being.

Our team (Craig *et al*^4^) systematically reviewed the effectiveness of CFT in clinical populations including depression, psychosis and borderline personality disorder. This included 15 studies (4 randomised controlled trials (RCTs)), finding significant improvements in symptomatology and self-compassion. CFT was acceptable and feasible to deliver in clinical settings, especially when delivered in a group format over at least 12 hours. The review concluded that CFT shows promise for a range of conditions, with RCT evidence urgently needed. We also (Craig *et al*^5^) developed and evaluated a 10-session manualised CFT intervention for dementia. This CFT case series (n=7) found clinically significant changes in depression (n=4) and anxiety (n=3), highlighting feasibility and potential. While this work^5^ focused on individual CFT sessions, there are strong economic and practical arguments for group delivery within the National Health Service (NHS).

This study aims to evaluate the feasibility, acceptability and fidelity of 12 sessions of group CFT as a treatment for people with dementia experiencing anxiety and/or depression. Blind assessments will be conducted at baseline, approximately 16 weeks and 6 months follow-up, to collect data on depression, anxiety, quality of life, quality of the carer–patient relationship, cognition, self-compassion and carer burden. Qualitative interviews will be used to gather participant, carer/supporter and clinician perspectives on the value, acceptability and feasibility of the intervention. Results of this study will determine if a full RCT is warranted.

## Methods and analysis

### Trial design

A single-blind feasibility RCT will compare group CFT plus treatment as usual (TAU) against TAU alone. 50 participants will be randomised to either the intervention group (CFT plus TAU) or control group (TAU). Randomisation will occur after baseline assessments are completed. Each arm will have approximately 25 participants, with up to 7 participants in each CFT group. The duration of the intervention will be 15 weeks, consisting of 12 hourly CFT therapy sessions. The 15-week block is being used to hold 12 sessions of CFT, to provide a buffer to allow for factors such as weather issues, therapist annual leave and illness, increasing the likelihood that participants will receive all 12 sessions. There will be assessments at baseline, during the 2 weeks prior to randomisation, at the end of the intervention at approximately 16 weeks and at 6 months follow-up.

To further inform our understanding of the process and feasibility of implementation, we will conduct semistructured, audiorecorded qualitative interviews with up to 20 participants with dementia, 15 carers/supporters and 10 NHS personnel (including group facilitators and service managers). These will provide insight into the feasibility of implementation and explore trial procedures from the perspectives of those who did and did not participate in the intervention or carers workshop and barriers to attendance.

### Study timelines

The study duration is 30 months, and the start date was 1 April 2023. Recruitment opened at NELFT on 8 November 2023 and we expect recruitment to be complete by the end of 2024 to allow time for the 6 months follow ups. Data cleaning and analysis will take place between April and October 2025.

### Study sponsor and monitoring

The study is sponsored by North East London NHS Foundation Trust who are responsible for the oversight of the study. The sponsor accepts responsibility for monitoring the trial across all participating sites and will conduct regular site visits to review the trial conduct, including participant recruitment, data collection and adherence to the protocol.

For any inquiries related to the study, please contact:

Victoria Dervish

Research Business Operations Manager

Research and Development Department

1^st^Floor Maggie Lilley Suite, Goodmayes Hospital, Barley Lane, Ilford, Essex, IG3 8XJ

Tel: 0300 555 1200 Ext. 64 478

Victoria.Dervish@nelft.nhs.uk

### Sample size

This is a feasibility study with no formal power calculation. Instead, a sufficient number of participants need to be recruited in order to determine the attrition and recruitment rates and how these are related to feasibility of a full-scale RCT. By setting our target sample size at 50, we will achieve adequate precision around our expected retention rate of 75% (95% CI 62% to 86%) to determine feasibility going forward. In addition to the sample size of 50 people with dementia, each site will be expected to recruit and consent carers/supporters into the trial, as they will be contributing to the quantitative data collection. We will conduct qualitative interviews with up to 20 people with dementia, up to 15 carers/supporters and up to 10 NHS personnel. Consent will be required before any qualitative interview can take place.

### Recruitment of participants

Participants are currently being recruited and enrolled to the study. Recruitment will primarily take place through the NHS Foundation Trusts of North East London, Oxford Health, Norfolk and Suffolk, the Black Country Healthcare, Central and North West London, Lincolnshire Partnership and Cheshire and Wirral Partnership.

Recruitment will be conducted in adherence with local Trust privacy notice permissions and consent to contact arrangements. The study will be promoted through relevant services and routes specific to the local Trust including non-NHS pathways such as third-sector organisations, supported living accommodation and care homes. We will recruit through ‘Join Dementia Research’, an online recruitment platform. ‘Join Dementia Research’ recruitment can only be from within the locality of the Trusts involved.

Participants will be approached by the recruiting team to discuss the study with the participant and their carer/supporter (where available and willing). If they are interested in taking part, both the participant and their carer/supporter (if applicable) will be provided with a relevant information sheet. A participant information sheet is included as online supplemental material file 1. If interested, the research team will arrange a meeting to answer questions and assess eligibility. If the participant and carer/supporter (if applicable) agree to take part, consent can be received either in person (in writing) or remotely (verbally). Two participant consent forms are provided as onlinesupplemental material files 2  3.

Inclusion criteria are as follows:

1. Meet Diagnostic and Statistical Manual of Mental Disorders, Fourth Edition for dementia of any type.^6^
2. Mild-to-moderate dementia as determined by the Clinical Dementia Rating Scale.^7^
3. Experience symptoms of depression and/or anxiety (≥8) as measured by the Hospital Anxiety and Depression Scale (HADS)^8^ or a minimum score of 5 and experience of depression and/or anxiety as reported by either the caregiver or clinician.
4. Have the capacity to consent to take part in research.
5. Can communicate in English.
6. Have access to WiFi, enabling them to partake in online CFT groups or the ability to attend a face-to-face group.
7. Are not participating in another interventional research programme concurrently.
8. Aged 18 and over.
9. People can be included whether or not they have a caregiver.

No age, care situation or access to teleconferencing devices exclusion criteria will be applied.

### Randomisation procedures and blinding

Participant randomisation will be undertaken remotely via a secure online system using a dynamic adaptive randomisation algorithm provided and maintained by the Clinical Trials Unit NWORTH, Bangor University^9^. Participants will have indicated whether they are able to attend face to face only, online only or can attend either session format (in addition to their preference) when they provide consent. From this, the researcher will construct ‘randomisation blocks’ based on their ability to attend either format. Once the recruiting site reaches approximately 10 recruits to a ‘randomisation block’ (ie, online or face to face), the randomisation procedure will be carried out. If there are too few participants at one site that can only attend online, then the online groups may be combined with other sites including participants from ‘Join Dementia Research’ to form one online group. The minimum number in each CFT group is 3 participants, with the maximum being 7. The results of the randomisation will be sent to the unblinded researcher and relayed to the participants, arrangements can then be made to begin the intervention or for TAU. The intervention should begin within a week of randomisation.

Due to the nature of the intervention, it is not possible to blind participants, however, researchers collecting outcome data will be blinded. As participants may accidentally unblind researchers during follow-up assessments, we will collect data on the occasions where this happens and where possible a different researcher will conduct future assessments. The trial statistician will remain blind throughout the duration of the study until the blinded analysis (detailed in the analysis plan) has been conducted and reported to the study team.

### Intervention

The intervention comprises 12 virtual or face-to-face group CFT sessions, each lasting 60 minutes, divided into three phases:

1. Phase 1: Introduction to CFT, psychoeducation on emotion regulation systems, formulation and goal setting.
2. Phase 2: Techniques for developing self-compassion, including imagery and writing compassionate letters to self.
3. Phase 3: Techniques to tolerate difficult feelings, focusing on maintaining benefits post intervention.

Sessions will include core CFT practices, for example, ‘soothing rhythm breathing’ and will introduce new concepts, such as the qualities of compassion and mindful awareness. Participants will reflect on the emotional experience of living with dementia, such as the ‘threat’ posed by the diagnosis to their sense of self and future, leading to fear, anxiety and disconnection. Sessions will conclude with suggestions for home practice, with participants receiving session summaries. CFT will be adapted to accommodate cognitive changes, with frequent repetition and the use of visual and verbal aids. Building on the experience of running Cognitive Stimulation Therapy (CST) groups,^10^ groups will consist of approximately five people and where possible there will be time for social interaction before and after the session.

Additional carer/supporter workshop: We will run a brief workshop (flexibility for online or face to face) for primary carers/supporters (if available) towards the beginning of the CFT programme. This workshop will educate carers/supporters on CFT principles, outline session content and offer tips for encouraging and supporting the therapy at home.

The intervention group will continue to have access to TAU (see description below). As both groups will have access to TAU, this study will look at the additional impact of CFT. For those who do not own a tablet and wish to complete the online intervention, 10 tablets will be purchased and lent to those participants, aiming to maximise inclusivity.

### Treatment as usual

TAU is defined as standard treatment available to people with dementia and depression and/or anxiety, which might include medication, other therapies, day care, input from health and social care professionals such as psychiatrists, psychologists and social workers or no treatment. We will collect information on all health and care services used by people with dementia (which we can compare with ongoing observational studies such as IDEAL^11^) to describe what TAU involves for each participant; this can be taken into account in a future, fully powered trial.

### Primary outcome measures

#### Feasibility outcomes

1. Feasibility of recruitment and retention, assessed by:

1.1. Successful recruitment of the target sample (50 people) in 14 months.

1.2. Retention rate of at least 75% of participants to 16-week follow-up.

2. Acceptability of the intervention, assessed by:

2.1. Overall attendance and retention rates among the CFT participants (over 60%).

2.2. Any negative or adverse events (AEs) related to the intervention.

2.3. Preference of virtual or face-to-face therapy.

3. Fidelity, assessed by:

3.1. Therapist completion of the fidelity checklist following each session.

3.2. Audio recording of all sessions and an independent researcher rating fidelity with a random 10% of the recordings. A total, mean fidelity score and percentage will be calculated for each CFT session. These scores will be compared across sites and providers. We will compare self-reports with observer ratings, providing some idea about the utility of self-reports in a future trial.

Feasibility of progression to a definitive RCT will be assessed on a ‘Stop/Review/Go’ basis, with a successful outcome being that all criteria are assessed as ‘Go’. Continuation will still be possible with a combination of ‘Review’ and ‘Go’ flags but will require additional discussion about how to proceed. Any ‘Stop’ criteria will indicate that either the design or processes will need to be overhauled and would indicate any future work would involve further piloting.

Indicative criteria would be:

- Recruitment of participants within 14 months: Go: ≥90%, review: 60%–89%, stop: <60%.
- Retention of participants at 6 months: Go: ≥75%, review: 45%–74%, stop: <45%.
- Number of sessions delivered: Go: ≥90%, review: 70%–89%, stop: ≤70%.
- Participant attendance at sessions delivered: Go: >80%, review: 50%–79%, stop: ≤50%.
- Collection of outcome data at a time point: Go: ≥80%, review 55%–79%, stop: <55%.

The flow of participants in the study will be summarised using a Consolidated Standards of Reporting Trials flow diagram. From this data, some of the primary outcome measures will be calculated, such as eligibility and retention rates and willingness to be randomised (randomisation/recruitment rate).

Acceptability of the intervention to clinicians and participants will be assessed both qualitatively and quantitively. Access to technology, ability to collect outcome data and retention of the intervention will be presented descriptively from data collected in the electronic case report forms (CRFs). Access to technology and availability to travel will be assessed as part of the initial screening session by the local researcher. The research team will record ability to collect outcome data and retention of the intervention.

Preliminary efficacy of the intervention will be assessed using the quantitative measures outlined below, all with good to excellent psychometric properties, by a researcher blind to group allocation, at week zero (baseline), approximately 16 weeks and 6 months. Suitability of secondary outcome measures for a definitive RCT will be established by analysis of completion and response rates, outcome sensitivity to change as a result of the intervention and qualitative findings as to the perceived benefits and their relative importance.

### Secondary outcome measures

As this is an unpowered feasibility study, we are not hypothesising significant changes in any secondary outcomes. However, we will explore changes in outcomes before and after the intervention, comparing the treatment and control group (TAU), and we may expect some positive trends. We are also exploring the differences between online and face-to-face groups and have no current hypothesis in terms of superiority.

### Quantitative evaluation

Data collected will be entered directly into a database hosted on REDCap by a member of the study team. REDCap is an internet cloud-based system with high-security data collection and management software. Assessments will be delivered virtually or face to face, depending on participant preference. Demographics and general information will be collected including age, gender, ethnicity, use of medication (including antidepressants, anxiolytics and cholinesterase inhibitors), treatment preference, participation in other activities and presence/absence of a carer/supporter. The baseline assessment will take approximately 90 minutes for the participant with dementia and approximately 25 minutes for the carer/supporter. We will pilot the assessment battery and if it is perceived as too arduous, we will revisit the assessments. We will ensure that we offer breaks during the assessment and hold more than one assessment session if required. There were no problems encountered conducting a similar battery assessment in our initial study.^5^ The following measures, all with good to excellent psychometric properties, will be collected by a researcher (blind to group allocation), at week zero (baseline).

### Measures to be completed by the participant

1. Mood
  - Cornell Scale for Depression in Dementia^12^: This scale measures symptoms of depression and rates depression in five categories including mood-related signs, behavioural disturbance and ideational disturbance, using self-rated reports from the person with dementia. Good reliability and validity have been demonstrated.
  - Rating Anxiety in Dementia^13^: This rates signs and symptoms of anxiety using interviews with people with dementia. There are 18 questions in 4 categories: worry, apprehension, vigilance, motor tension and autonomic hypersensitivity. A score of 11 or above indicates significant clinical anxiety. It has good inter-rater and test–retest reliability and is sensitive to change.
2. Quality of life
  - The Dementia Quality of Life (DEMQOL) instrument is included because quality of life has been linked to mood in dementia. It measures five domains of quality of life; health and well-being, cognitive functioning, social relationships and self-concept. The scale uses self-rated reports of quality of life from the person with dementia. It has high internal consistency (0.87) and acceptable inter-rater reliability (intracluster correlation, ICC 0.84)^14^
  - The EuroQol 5-Dimension 5-Level (EQ-5D-5L)^15^ is included because quality of life has been linked to mood in dementia. It measures five domains of the participant’s health-related quality of life (mobility, self-care, usual activities, pain/discomfort and anxiety/depression). The scale uses self-rated reports of quality of life from the person with dementia. It has good internal consistency and inter-rater and test–retest reliability.
  - Two quality-of-life measures are used because EQ-5D-5L supports quality-adjusted life-years (QALYs) calculations in health economic evaluations, while DEMQOL is recommended by National Institute for Health and Care Excellence (NICE) guidelines for assessing quality of life in dementia patients.
3. Cognitive function
  - The Montreal Cognitive Assessment^16^ will enable us to explore whether mood is a predictor of cognitive change. It is a 30-point test consisting of 13 tasks covering 8 domains: visuospatial/executive functions, naming, verbal memory registration and learning, attention, abstraction, delayed verbal memory, and orientation. It has demonstrated high sensitivity and specificity.
4. Self-compassion
  - The Short-Self-Compassion Scale^17^ measures self-kindness, self-judgement, common humanity, isolation, mindfulness and overidentification. It has good internal consistency (α≥0.86), factorial validity and convergent validity. It has not been specifically validated for use in dementia populations although was completed successfully in our pilot study.^5^ We will look at the validation data within our analysis.
5. Relationship with caregiver
  - The Quality of Caregiver and Patient Relationship (QCPR) scale^18^ is a 14-item scale measuring relationship quality, it has displayed good psychometric properties and internal consistency, with a Cronbach’s alpha of 0.82. For those who have a caregiver, it will be rated by both the person with dementia and their caregiver enabling both perspectives to be examined.
6. Resource use
  - The Client Service Receipt Inventory (CSRI)^19^ is used extensively in economic studies of dementia: it gathers data on accommodation, medication, use of public, private and voluntary sector services and inputs from carers/supporters.

### Measures to be completed by the carer/supporter (if applicable)

1. Relationship with caregiver
  - As described above, the QCPR scale^18^ will be implemented to gain the caregivers’ perspective.
2. Caregiver burden
  - The revised Zarit Burden Interview (ZBI)^20^ consists of 22 items rated on a 5-point Likert scale that ranges from 0 (never) to 4 (nearly always) with the sum of scores ranging between 0 and 88. Higher scores indicate greater burden. The ZBI is used extensively to assess caregiving burden in clinical and research settings.
3. Quality of Life
  - The EQ-5D-5L proxy will be used to calculate QALYs in line with NICE guidance.^15^ The DEMQOL-proxy will also be administered.

### Qualitative evaluation

Group facilitators will complete an attendance register and a fidelity checklist to determine if the intervention is delivered and adhered to as intended. All sessions will be audio recorded, and an independent researcher will rate fidelity with a random 10% of the recordings. We will conduct semistructured, audio recorded interviews with up to 20 participants with dementia (including up to 10 control group participants with dementia, to explore trial and intervention procedures from the perspective of those who did not participate in the intervention). We will also interview up to 15 carers/supporters (including those who did not attend the workshop to better explore barriers to attendance). We will approach and plan to interview up to 10 NHS personnel (including group facilitators and service managers) for qualitative interviews to determine feasibility of implementation.

Interview topic guides will be guided by process evaluation parameters described in recognised frameworks^21 22^ and draw on theoretical models such as normalisation process theory.^23^ Interview topic guides will be coproduced with people with dementia and their carers/supporters.

Analysis will follow Braun and Clarke’s methods of thematic analysis^24^ and will be done using NVivo. This analysis will reveal the experiences of CFT and its delivery, the barriers and facilitators to its uptake and continued use, and the perceived benefits for the person participating in CFT and how were these realised (mechanism of change). Findings will also be evaluated with a focus on implementation considerations.

### Discontinuation/withdrawal of participants

A participant may be withdrawn from the trial whenever continued participation is no longer in the participant’s best interests, but the reasons for doing so should be recorded. Reasons for discontinuing the trial may include:

- Disease progression while in trial.
- Chronic current illness.
- Patients withdrawing consent or losing capacity to consent.

The decision of a participant to withdraw from treatment will be recorded in the electronic CRF and medical notes. If a participant withdraws from the intervention, they will be asked to continue to provide follow-up data. Their decisions regarding withdrawal from the intervention and withdrawal from follow-up will be recorded in their medical notes and in the trial electronic CRF, along with any reasons that they have shared.

### Recording and reporting of serious AEs and AEs

There are no known adverse effects of CFT. However, taking part may possibly cause distress/inconvenience for some participants with dementia. It is of particular importance in this trial to capture events related to the procedure (CFT). The assessment of a possible relationship of a serious AE or AE with trial procedures will be recorded and reported as part of the trial to ensure it is safe.

### Statistical analysis

A statistical analysis plan will be written, agreed and signed off before data lock is complete detailing all quantitative analysis to be conducted by NWORTH. Primary analysis will be based on the feasibility outcomes defined above. For the proposed clinical outcomes, we will conduct exploratory statistical analyses using ‘intention-to-treat’ principles. The focus of the results will be on the estimates of the treatment effects rather than statistical significance. Therefore, differences between the two comparison groups will be presented in the form of an unadjusted mean difference for continuous outcomes, and an OR for binary outcomes, with their associated 95% CIs. Given the nature of this study, no imputation methods will be used over and above any rules indicated by the measures for handling missing items. Missing data will be used to assess the suitability of the measure’s future use. Despite individual randomisation, the treatment is group based, and therefore, we will estimate the potential ICC coefficient. This, together with indications from other work, will guide the sample size of a future trial.

### Health economic analysis

We will calculate the cost per participant for the CFT intervention, including training costs for group leads and travel costs for participants. We will also undertake some preliminary analyses to inform the economic evaluation that would accompany a full trial. This will be based on the premise that we will wish to estimate the incremental cost per QALY gained from the intervention versus control, and also the incremental cost per change in primary outcome. Consequently, we will collect data on the use of services (CSRI) and health-related quality of life (DEMQOL and EQ-5D-5L) and examine overall response rates and completion rates of individual questions.

### Patient and public involvement

Patients and the public have been involved since the project inception. The current proposal was developed in collaboration with our patient and public involvement (PPI) lead who attends most monthly management meetings and will be invited to coauthor publications and copresent to diverse audiences.

Our PPI advisory group consists of four people with various types of dementia, one of whom is from a South Asian community, and one family caregiver. They will meet at least four times including the manual adaptation, modifying the CSRI, devising the qualitative interviews and dissemination stages to ensure clarity for a lay audience. Our trial steering committee (TSC) also includes a family caregiver from a South Asian community, they will provide cultural insights to ensure the feasibility trial is relevant to South Asian communities.

### Ethics and dissemination

This study was approved by the London Riverside Research Ethics Committee and Health Research Authority (Ref: 23/LO/0535) in August 2023. Further ethical approval will be sought from HRA if any amendments to the protocol are needed. All changes will be communicated to the relevant trial sites and principal investigators. A TSC has been appointed to provide independent study oversight and will meet at least three times throughout the duration of the study.

Dissemination plans include feedback to all participating service users, publishing findings through NELFT and partnering trusts websites and newsletters, working with UCL media to develop appropriate press releases and social media communications, peer-reviewed publications, publications in relevant professional and education journals, conference presentations and disseminating through partner organisations. Data from the trial will be shared on reasonable request.

## supplementary material

10.1136/bmjopen-2024-093249 online supplemental file 1

10.1136/bmjopen-2024-093249 online supplemental file 2

10.1136/bmjopen-2024-093249 online supplemental file 3
