# Supplementary material for: Being kind to ourselves: group compassion-focused therapy (CFT) versus treatment as usual (TAU) to improve depression and anxiety in dementia – a protocol for a mixed-methods feasibility randomised controlled trial within the NHS
Source: BMJ Open. 2024 Dec 3;14(12):e093249. doi: 10.1136/bmjopen-2024-093249 (PMC11628998; doi:10.1136/bmjopen-2024-093249)
Supplement: online supplemental file 1 [file bmjopen-14-12-s001.pdf]

PARTICIPANT INFORMATION SHEET

Study Title: Being kind to ourselves: A feasibility randomised controlled trial of Compassion Focused Therapy (CFT) to improve depression and anxiety in Dementia.

Invitation to participate in a research study

You are being invited to take part in a research study. Before you decide, it is important for you to understand why the research is being done and what it will involve. Please take time to read the following information carefully and discuss it with others if you wish. Thank you for reading this information sheet.

What is the purpose of the study?

This study aims to find out whether Compassion Focused Therapy, delivered in a group format online or face-to-face, can help improve mood, anxiety, quality of life, cognition, relationship with carer/supporter and self-compassion of people experiencing dementia and depression or anxiety.

What is Compassion Focused Therapy?

Compassion Focused Therapy helps us to develop kindness to ourselves when we are in distress. When we are unhappy we may become self-critical about how we are coping, or mentally challenge ourselves about things we have done wrong in life. Developing compassion towards ourselves and others may reduce stress and low mood and increase our wellbeing.

Research has shown Compassion Focused Therapy to be helpful for people experiencing a range of difficulties, especially those who experience shame and self-criticism. Research has also shown that people who have higher levels of self-compassion in older age are happier. Memory problems can make us feel low and at times criticize ourselves. Therefore, this study is designed to find out if people with dementia attending Compassion Focused Therapy experience improvements in their mood, anxiety, quality of life and self-compassion.

This study is a feasibility trial, this means that results from this trial will determine if a full randomised controlled trial is warranted. The trial will look at things like how many people are suitable to take part, how easily we can recruit, dropout rates, and the cost of delivering Compassion Focused Therapy. All these will help us determine the 'acceptability' of Compassion Focused Therapy as an online or face-to-face intervention for people with dementia.

What happens in Compassion Focused Therapy?

If you choose to take part, you will be randomly assigned to either the Compassion Focused Therapy group or a 'control' group. There is an equal, 50/50 chance of you being in either group. If you are in the control group, you will not receive any additional treatment.

If you are randomly allocated to the intervention group, you will be invited to attend twelve, 60-minute online or face-to-face small group Compassion Focused Therapy sessions. These will occur once a week for twelve weeks. The sessions will involve meeting with a clinical professional and other people with dementia to discuss topics such as low mood, memory problems, and coping mechanisms. During the sessions you will also do activities such as gentle breathing and self-compassion exercises. There will be time to reflect as a group on the emotional experience of living with dementia. Sessions will end with suggesting home practices, with participants given session summaries. There will be time for social interaction before and after the session, either over a video conference platform or face-to-face.

If you are randomised to the Compassion Focused Therapy group, we will run a brief workshop for your carer / supporter (if applicable) around the beginning of the Compassion Focused Therapy program. This will provide information on the principles of Compassion Focused Therapy, an outline of what we intend to do in sessions and tips on what can be done at home to support the therapy.

Regardless of which group you are allocated to (Compassion Focused Therapy or control), you will continue to have access to your usual care, including input from health and social care professionals, dementia medication and your usual day activities.

Why have I been invited to take part?

You have been invited to take part because you recognize that you are experiencing dementia and difficulties with your mood.

Do I have to take part?

It is up to you to decide whether or not to take part. If you do decide to take part, you will be given this information sheet to keep and be asked to sign a consent form.

You can still take part in the study if you do not have a carer / supporter who can attend, or if you decide that you would prefer to attend alone.

What will happen to me if I take part?

Following discussion of any questions you may have with a researcher, and signing the consent form, all participants will be asked to:

- Meet with a researcher for approximately 1.5 hours to answer questions about your mood, anxiety, quality of life, and thinking. If applicable, we will also invite a carer / supporter to attend this meeting with you.
- Meet with a researcher again after the 12 sessions to answer the same questions as before, and again 6 months after your initial assessment. Each follow up will take approximately 1.5 hours.

Additionally, IF you have been randomised to receive Compassion Focused Therapy, you will be invited to:

- Attend twelve, weekly 1- hour small group Compassion Focused Therapy sessions either online or face-to-face.

Optional interviews:

You may be randomly selected to attend an interview to discuss your experience of taking part in the study. Your carer / supporter (if applicable) may be asked to attend an interview separately to discuss their experience of supporting you.

What do I have to do?

You can carry on your everyday activities as normal while participating in the study. All we ask is that if you are randomly allocated to the intervention group, that you try to attend all twelve sessions. We understand there may be times when you are unable to attend a session.

What are the possible disadvantages and risks of taking part?

We appreciate that when you are experiencing memory problems, it may be hard to talk about things like your mood and quality of life. The researchers carrying out the assessment, intervention, and interview have clinical experience and are working under supervision.

You will be encouraged but never forced to take part in a particular activity during the sessions.

Overall, the risks of taking part in this study are minimal. However, some people find that certain types of therapy do not help them or make them feel worse. If you find participating in the study distressing, let us know and we can try to resolve the difficulty together or discuss other options of support. You

are free to withdraw from the study at any point.

If you lose capacity to consent, you will be withdrawn from the study and no further data will be collected, however data collected up until that point will be retained for use in the study. Withdrawing from the study will not affect the standard of care you receive.

What are the possible benefits of taking part?

If you do decide to take part in the study and are allocated to the intervention group, we hope that your attendance at the sessions is a helpful experience for you. Previous research into compassion suggests that people can experience greater awareness, acceptance, control, improved coping and wellbeing. Regardless of whether you receive the intervention or not, the information we get from this study may help us to support people with dementia and their carers / supporters better in future.

How will we use information about you?

We will need to use information from you for this research project. This information will include your name and contact details. People will use this information to do the research or to check your records to make sure that the research is being done properly. People who do not need to know who you are will not be able to see your name or contact details. Your data will have a code number instead. We will ask for your permission to send your GP a letter explaining that you will be taking part in the study.

We will keep all information about you safe and secure.

Once we have finished the study, we will keep some of the data so we can check the results. We will write our reports in a way that no-one can work out that you took part in the study.

The Compassion Focused Therapy sessions will be audio recorded for research purposes with consent from both parties and will be kept password protected. Any data transferred across the team will be encrypted, password protected and contain no identifiable data. At the end of the trial, all essential documentation will be archived securely by the study Sponsor for a minimum of 5 years from the declaration of end of trial.

What are your choices about how your information is used?

- You can stop being part of the study at any time, without giving a reason, but we will keep information about you that we already have.
- We need to manage your records in specific ways for the research to be reliable. This means that we won't be able to let you see or change the data we hold about you.

Where can you find out more about how your information is used?

You can find out more about how we use your information:

- At www.hra.nhs.uk/information-about-patients/
- Our leaflet available from www.hra.nhs.uk/patientdataandresearch
- By asking one of the research team.
- By sending an email to NELFT Data Protection Officer, Robert Paley, Robert.Paley@nelft.nhs.uk.
- By ringing us on 0300 300 1748.

What if something goes wrong?

If you have a concern about any aspect of this study, you should ask to speak to the researchers who will do their best to answer your questions [site specific contact information]. If you remain unhappy and wish to complain formally, you can do this by contacting [site specific NHS PALS contact information].

Every care will be taken in the course of this study. However, in the unlikely event that you are injured by taking part, compensation may be available. In the event that something does go wrong, and you are harmed during the research, and this is due to someone's negligence then you may have grounds for a legal action for compensation against North East London NHS Foundation Trust but you may have to pay your legal costs. The normal National Health Service complaints mechanisms will still be available to you (if appropriate).

Regardless of this, if you wish to make a complaint about any aspect of the way you have been approached or treated during the course of this study or if you are unhappy with anything about your participation, you can contact Professor Aimee Spector who is the Chief Investigator for the research and is based at University College London (UCL):

Professor Aimee Spector
Department of Clinical, Educational and Health Psychology
UCL
Gower Street
WC1E 6BT
Email: a.spector@ucl.ac.uk
Tel: 0207 679 1844

Who is organising and funding the research?

The research is being organized by Professor Aimee Spector at University College London (UCL). The study is being sponsored by North East London NHS Foundation Trust. The project is funded by the

National Institute for Health and Care Research (Award ID: NIHR203524). The National Institute for Health and Care Research will not be involved in the conduct of the study.

What will happen to the results of the research?

The study will be registered on a public web-based database where the study design and results can be viewed. The results of the trial will also be published in a scientific journal and presented at conferences, but you will not be identified. Once the study has ended, you can meet with a researcher to find out about the results, a written summary of the findings can also be requested.

Who has reviewed the study?

All research in the NHS is looked at by an independent group of people, called a Research Ethics Committee, to protect your interests. This study has been reviewed and given favourable opinion by London Riverside Research Ethics Committee.

Who can I contact for further information?

For more information about this research, please contact: [site specific contact information]

Independent Advice

If you would like to speak to someone about this research who is independent from the research team, please contact Sandeep Toot using the following details:

Dr Sandeep Toot (Deputy Director, Research & Development)
NELFT Research & Development Department
1st Floor Maggie Lilley Suite
Goodmayes Hospital
Barley Lane, Goodmayes
Essex, IG3 8XJ
Phone: 0300 555 1200 (dial extension 64453) Email: Sandeep.toot@nelft.nhs.uk

Thank you for thinking about taking part in this research study.
