# Supplementary material for: Being kind to ourselves: group compassion-focused therapy (CFT) versus treatment as usual (TAU) to improve depression and anxiety in dementia – a protocol for a mixed-methods feasibility randomised controlled trial within the NHS
Source: BMJ Open. 2024 Dec 3;14(12):e093249. doi: 10.1136/bmjopen-2024-093249 (PMC11628998; doi:10.1136/bmjopen-2024-093249)
Supplement: online supplemental file 2 [file bmjopen-14-12-s002.pdf]

UCL

[INSERT TRUST LOGO]

PARTICIPANT CONSENT FORM – QUALITATIVE INTERVIEW

Title of project: Being kind to ourselves: A feasibility randomised controlled trial of Compassion Focused Therapy (CFT) to improve depression and anxiety in Dementia.

Centre Number:

Please Initial Boxes:
(researcher initials if verbal consent)

I have been asked to participate in an interview about my experience in the Compassion Focused Therapy research study. I have had the opportunity to ask questions about the study and have read the information sheet [date, version] and understand what is involved.	
I understand that the session will be audio recorded for the purpose of research.	
I understand that if I lose capacity to consent, that I will be withdrawn from the study and no further data will be collected, however data collected up until that point will be retained for use in the study.	
I understand that I can request to withdrawn from the study at any time, without giving any reason.	
I understand that any quotations used from the recording will be anonymised to ensure that I cannot be identified.	
I consent to taking part in the interview section of this research study.	

Name of participant | **Date (DD/MMM/YYYY)** | **Signature of participant**

Signature of researcher (verbal consent)

Name of researcher | **Date (DD/MMM/YYYY)** | **Signature of researcher**

1 copy for participant, 1 stored in research file, 1 stored in medical records.
