# Supplementary material for: Being kind to ourselves: group compassion-focused therapy (CFT) versus treatment as usual (TAU) to improve depression and anxiety in dementia – a protocol for a mixed-methods feasibility randomised controlled trial within the NHS
Source: BMJ Open. 2024 Dec 3;14(12):e093249. doi: 10.1136/bmjopen-2024-093249 (PMC11628998; doi:10.1136/bmjopen-2024-093249)
Supplement: online supplemental file 3 [file bmjopen-14-12-s003.pdf]

UCL

[INSERT TRUST LOGO]

CONSENT FORM FOR PARTICIPANT

Study Title: Being kind to ourselves: A feasibility randomised controlled trial of Compassion Focused Therapy (CFT) to improve depression and anxiety in Dementia.

Centre Number:

Please Initial Boxes:
(researcher initials if verbal consent)

I confirm that I have read and understand the participant information sheet dated [date, version] for the above study, have had the opportunity to ask questions and have had these answered acceptably.	
I understand that my participation is voluntary and that I am free to withdraw at any time, without giving any reason, without my medical care or legal rights being affected.	
I understand that if I lose capacity to consent, that I will be withdrawn from the study and no further data will be collected, however data collected up until that point will be retained for use in the study.	
I understand that sections of any of my medical notes and data collected during the study may be looked at by individuals involved in the study, where it is relevant to my taking part in this research. I give my permission for these individuals to have access to my records	
I give permission for my GP to be informed of my participation in the study	
I understand that all information given by me or about me will be treated as confidential by the research team.	
I understand that I will be randomly assigned to either the Compassion Focused Therapy group or a 'control' group. I understand that if I am assigned to the control group, that I will not receive Compassion Focused Therapy.	
I consent to the audio recording of the intervention sessions for research purposes.	
I agree to take part in the above study.	

Name of participant **Date (DD/MMM/YYYY)** **Signature of participant**

Signature of researcher (verbal consent)

Name of researcher **Date (DD/MMM/YYYY)** **Signature of researcher**

1 copy for participant, 1 stored in research file, 1 stored in medical records.
