# Supplementary material for: Being kind to ourselves: group compassion-focused therapy (CFT) versus treatment as usual (TAU) to improve depression and anxiety in dementia – a protocol for a mixed-methods feasibility randomised controlled trial within the NHS
Source: BMJ Open. 2024 Dec 3;14(12):e093249. doi: 10.1136/bmjopen-2024-093249 (PMC11628998; doi:10.1136/bmjopen-2024-093249)
Supplement: Supplementary file 4 [file bmjopen-2024-093249.reviewer_comments.pdf]

PEER REVIEW HISTORY

BMJ Open publishes all reviews undertaken for accepted manuscripts. Reviewers are asked to complete a checklist review form and are provided with free text boxes to elaborate on their assessment. These free text comments are reproduced below.

ARTICLE DETAILS

Title (Provisional)

Being kind to ourselves: Group Compassion Focused therapy (CFT) versus Treatment as Usual (TAU) to improve depression and anxiety in dementia; a protocol for a mixed-methods feasibility randomised controlled trial within the NHS.

Authors

Spector, Aimee; Melville, Mel; Henderson, Catherine; Knapp, Martin; Oliver, Keith; Robinson, Louise; Royan, Lindsay; Stott, Joshua; Williams, Lisa; Evans, Rachel

---

VERSION 1 - AUTHOR RESPONSE

n/a
